# Supplementary material for: Association between Proton Pump Inhibitor Use and Parkinson’s Disease in a Korean Population
Source: Pharmaceuticals (Basel). 2022 Mar 9;15(3):327. doi: 10.3390/ph15030327 (PMC8955848; doi:10.3390/ph15030327)
Supplement: Supplementary file 1 [file pharmaceuticals-15-00327-s001.zip › pharmaceuticals-1620193-supplementary.pdf]

Table S1. Adjusted odds ratios of proton pump inhibitor use for Parkinson's disease with subgroup analyses stratified based on various comorbidities

Characteristics	Number of Parkinson's diseases	Number of controls	Odds ratio for Parkinson's disease (95% confidence interval)		P-value for interaction
	(exposure/total, %)	(exposure/total, %)	Adjusted[b]	P-value	
Age groups					0.151
Age <75 years old (n = 18,470)	610/3,694 (16.5%)	1,669/14,776 (11.3%)	1.25 (1.11-1.40)	<0.001[a]	
Age ≥75 years old (n = 11,495)	419/2,299 (18.2%)	1,158/9,196 (12.6%)	1.36 (1.19-1.55)	<0.001[a]	
Sex					0.589
Male (n = 14,000)	422/2,800 (15.1%)	1,257/11,200 (11.2%)	1.21 (1.06-1.38)	0.005[a]	
Female (n = 15,965)	607/3,193 (19.0%)	1,570/12,772 (12.3%)	1.36 (1.21-1.53)	<0.001[a]	
Income groups					0.208
Low (n = 13,085)	478/2,617 (18.3%)	1,201/10,468 (11.5%)	1.43 (1.26-1.63)	<0.001[a]	
High (n = 16,880)	551/3,376 (16.3%)	1,626/13,504 (12.0%)	1.20 (1.07-1.35)	0.002[a]	
Region of residence					0.091
Urban (n = 11,120)	375/2,224 (16.9%)	970/8,896 (10.9%)	1.43 (1.23-1.65)	<0.001[a]	
Rural (n = 18,845)	654/3,769 (17.4%)	1,857/15,076 (12.3%)	1.23 (1.11-1.38)	<0.001[a]	
Obesity					0.662
Underweight (n = 1,183)	51/251 (20.3%)	114/932 (12.2%)	1.57 (1.04-2.38)	<0.001[a]	
Normal (n = 10,805)	355/2,141 (16.6%)	1,008/8,664 (11.6%)	1.23 (1.06-1.42)	0.006[a]	
Overweight (n = 7,819)	269/1,564 (17.2%)	769/6,255 (12.3%)	1.23 (1.04-1.45)	0.016[a]	
Obese (n = 10,158)	354/2,037 (17.4%)	936/8,121 (11.5%)	1.33 (1.15-1.54)	<0.001[a]	
Smoking status					0.547
Nonsmokers (n = 22,836)	810/4,733 (17.1%)	2,162/18,103 (11.9%)	1.26 (1.14-1.39)	<0.001[a]	
Past and current smokers (n = 7,129)	219/1,260 (17.4%)	665/5,869 (11.3%)	1.35 (1.12-1.62)	0.001[a]	
Alcohol consumption					0.074
<1 time a week (n = 17,400)	792/4,680 (16.9%)	1,917/17,436 (11.0%)	1.34 (1.21-1.48)	<0.001[a]	
≥1 time a week (n = 7,849)	237/1,313 (18.1%)	910/6,536 (13.9%)	1.10 (0.92-1.30)	0.306	
Blood pressure					0.434
SBP <140 mmHg and DBP <90 mmHg (n = 20,203)	725/3,978 (18.2%)	2,064/16,225 (12.7%)	1.26 (1.13-1.39)	<0.001[a]	
SBP ≥140 mmHg and DBP ≥90 mmHg (n = 9,762)	304/2,015 (15.1%)	763/7,747 (9.8%)	1.33 (1.13-1.55)	0.001[a]	
Fasting blood glucose					0.011[a]
<100 mg/dL (n = 17,400)	513/3,235 (15.9%)	1,618/14,165 (11.4%)	1.20 (1.07-1.36)	0.003[a]	
≥100 mg/dL (n = 12,565)	516/2,758 (18.7%)	1,209/9,807 (12.3%)	1.36 (1.20-1.54)	<0.001[a]	
Total cholesterol level					0.118
<200 mg/dL (n = 16,447)	574/3,369 (17.0%)	1,653/13,078 (12.6%)	1.18 (1.05-1.33)	0.004[a]	
≥200 mg/dL (n = 13,518)	455/2,624 (17.3%)	1,174/10,894 (10.8%)	1.40 (1.23-1.60)	<0.001[a]	
Charlson Comorbidity Index					0.016[a]
0 (n = 15,930)	367/2,376 (15.4%)	1,402/13,554 (10.3%)	1.29 (1.12-1.48)	<0.001[a]	

1 (n = 5,760)	228/1,369 (16.7%)	588/4,391 (13.4%)	1.11 (0.93-1.34)	0.255	
≥2 (n = 8,275)	434/2,248 (19.3%)	837/6,027 (13.9%)	1.32 (1.14-1.52)	<0.001[a]	
Gastroesophageal reflux disease					0.286
No (n = 24,895)	395/4,746 (8.3%)	1,109/20,149 (5.5%)	1.36 (1.20-1.54)	<0.001[a]	
Yes (n = 5,070)	634/1,247 (50.8%)	1,718/3,823 (44.9%)	1.27 (1.12-1.45)	<0.001[a]	
H2 blocker use					0.028[a]
Nonusers (n = 10,069)	110/1,545 (7.1%)	498/8,524 (5.8%)	1.15 (0.90-1.46)	0.268	
Users (n = 19,896)	919/4,448 (20.7%)	2,329/15,448 (15.1%)	1.29 (1.18-1.42)	<0.001[a]	
A history of head trauma					<0.001[a]
No (n = 28,671)	955/5,516 (17.3%)	2,727/23,155 (11.8%)	1.29 (1.18-1.40)	<0.001[a]	
Yes (n = 1,294)	74/477 (15.5%)	100/817 (12.2%)	1.15 (0.78-1.68)	0.486	
A history of other degenerative diseases of the nervous system					<0.001[a]
No (n = 28,933)	948/5,517 (17.2%)	2,756/23,416 (11.8%)	1.27 (1.17-1.39)	<0.001[a]	
Yes (n = 1,032)	81/476 (17.0%)	71/556 (12.8%)	1.28 (0.86-1.89)	0.221	

Note: DBP–Diastolic blood pressure; SBP–Systolic blood pressure. [a] Conditional or unconditional logistic regression analysis, Significance at P <0.05; [b] Adjusted for age, sex, income, region of residence, blood pressure, fasting blood glucose, total cholesterol level, obesity, smoking, alcohol consumption, the Charlson comorbidity index, gastroesophageal reflux disease, H2 blocker use, and a history of head trauma or other degenerative diseases of the nervous system.

Table S2. Adjusted odds ratios of proton pump inhibitor use for ≥30 days compared to proton pump inhibitor use for <30 days with subgroup analyses stratified by covariates

Characteristics	Number of Parkinson's diseases	Number of controls	Odds ratios for Parkinson's disease (95% confidence interval)		P-value for interaction
	(exposure/total, %)	(exposure/total, %)	Adjusted[b]	P-value	
Age groups					0.316
Age <75 years old (n = 18,470)	477/3,694 (12.9%)	1,197/14,776 (8.1%)	1.31 (1.15-1.49)	<0.001[a]	
Age ≥75 years old (n = 11,495)	360/2,299 (15.7%)	898/9,196 (9.8%)	1.49 (1.29-1.73)	<0.001[a]	
Sex					0.396
Male (n = 14,000)	350/2,800 (12.5%)	956/11,200 (8.5%)	1.27 (1.10-1.47)	0.001[a]	
Female (n = 15,965)	487/3,193 (15.3%)	1,139/12,772 (8.9%)	1.48 (1.30-1.69)	<0.001[a]	
Income groups					0.167
Low (n = 13,085)	390/2,617 (14.9%)	892/10,468 (8.5%)	1.56 (1.35-1.80)	<0.001[a]	
High (n = 16,880)	447/3,376 (13.2%)	1,203/13,504 (8.9%)	1.26 (1.10-1.44)	0.001[a]	
Region of residence					0.478
Urban (n = 11,120)	290/2,224 (13.0%)	718/8,896 (8.1%)	1.45 (1.22-1.71)	<0.001[a]	
Rural (n = 18,845)	547/3,769 (14.5%)	1,377/15,076 (9.1%)	1.36 (1.21-1.54)	<0.001[a]	
Obesity					0.621
Underweight (n = 1,183)	44/251 (17.5%)	79/932 (8.5%)	2.14 (1.37-3.34)	0.001[a]	
Normal (n = 10,805)	284/2,141 (13.3%)	734/8,664 (8.5%)	1.25 (1.06-1.48)	0.009[a]	
Overweight (n = 7,819)	226/1,564 (14.5%)	546/6,255 (8.7%)	1.45 (1.20-1.75)	<0.001[a]	
Obese (n = 10,158)	283/2,037 (13.9%)	736/8,121 (9.1%)	1.33 (1.12-1.56)	0.001[a]	
Smoking status					0.945
Nonsmokers (n = 22,836)	657/4,733 (13.9%)	1,584/18,103 (8.7%)	1.35 (1.21-1.51)	<0.001[a]	
Past and current smokers (n = 7,129)	180/1,260 (14.3%)	511/5,869 (8.7%)	1.40 (1.14-1.72)	0.001[a]	
Alcohol consumption					0.523
<1 time a week (n = 17,400)	639/4,680 (13.7%)	1,442/17,436 (8.3%)	1.39 (1.24-1.55)	<0.001[a]	
≥1 time a week (n = 7,849)	198/1,313 (15.1%)	653/6,536 (10.0%)	1.27 (1.05-1.54)	0.015[a]	
Blood pressure					0.752
SBP <140 mmHg and DBP <90 mmHg (n = 20,203)	602/3,978 (15.1%)	1,544/16,225 (9.5%)	1.37 (1.22-1.53)	<0.001[a]	
SBP ≥140 mmHg and DBP ≥90 mmHg (n = 9,762)	235/2,015 (11.7%)	551/7,747 (7.1%)	1.35 (1.13-1.62)	0.001[a]	
Fasting blood glucose					0.085
<100 mg/dL (n = 17,400)	418/3,235 (12.9%)	1,177/14,165 (8.3%)	1.31 (1.14-1.50)	<0.001[a]	
≥100 mg/dL (n = 12,565)	419/2,758 (15.2%)	918/9,807 (9.4%)	1.42 (1.23-1.63)	<0.001[a]	
Total cholesterol level					0.909
<200 mg/dL (n = 16,447)	489/3,369 (14.5%)	1,230/13,078 (9.4%)	1.35 (1.19-1.53)	<0.001[a]	
≥200 mg/dL (n = 13,518)	348/2,624 (13.3%)	865/10,894 (7.9%)	1.37 (1.18-1.59)	<0.001[a]	
Charlson Comorbidity Index					0.028[a]

0 (n = 15,930)	287/2,376 (12.1%)	999/13,554 (7.4%)	1.34 (1.14-1.57)	<0.001[a]	
1 (n = 5,760)	181/1,369 (13.2%)	430/4,391 (9.8%)	1.23 (1.00-1.51)	0.050	
≥2 (n = 8,275)	369/2,248 (16.4%)	666/6,027 (11.1%)	1.40 (1.20-1.63)	<0.001 [a]	
Gastroesophageal reflux disease					0.076
No (n = 24,895)	302/4,746 (6.4%)	746/20,149 (3.7%)	1.52 (1.32-1.76)	<0.001[a]	
Yes (n = 5,070)	535/1,247 (42.9%)	1,349/3,823 (35.3%)	1.34 (1.17-1.54)	<0.001[a]	
H2 blocker use					0.001[a]
Nonusers (n = 10,069)	87/1,545 (5.6%)	379/8,524 (4.4%)	1.13 (0.86-1.49)	0.368	
Users (n = 19,896)	750/4,448 (16.9%)	1,716/15,448 (11.1%)	1.39 (1.25-1.54)	<0.001[a]	
A history of head trauma					<0.001[a]
No (n = 28,671)	771/5,516 (14.0%)	2,023/23,155 (8.7%)	1.35 (1.22-1.49)	<0.001[a]	
Yes (n = 1,294)	66/477 (13.8%)	72/817 (8.8%)	1.62 (1.06-2.47)	0.024[a]	
A history of other degenerative diseases of the nervous system					<0.001[a]
No (n = 28,933)	770/5,517 (14.0%)	2,036/23,416 (8.7%)	1.37 (1.24-1.51)	<0.001[a]	
Yes (n = 1,032)	67/476 (14.1%)	59/556 (10.6%)	1.22 (0.79-1.87)	0.369	

Note: DBP–Diastolic blood pressure; SBP–Systolic blood pressure. [a] Conditional or unconditional logistic regression analysis, Significance at P <0.05; [b] Adjusted for age, sex, income, region of residence, blood pressure, fasting blood glucose, total cholesterol level, obesity, smoking, alcohol consumption, the Charlson comorbidity index, gastroesophageal reflux disease, H2 blocker use, and a history of head trauma or other degenerative diseases of the nervous system.

Table S3. Adjusted odds ratios for Parkinson's disease per 90 days of proton pump inhibitor use with subgroup analyses stratified by covariates

Characteristics	Mean duration of PPI use in Parkinson's disease (Standard deviation)	Mean duration of PPI use in control (Standard deviation)	Odds ratios for Parkinson's disease (95% confidence interval)		P-value for Interaction
			Adjusted[b]	P-value	
Age <75 years old (n = 18,470)					First-generation PPIs: 0.173 Second-generation PPIs: 0.049[a]
First-generation PPIs	10.50 (40.87)	6.04 (30.48)	1.16 (1.06-1.27)	0.002[a]	
Second-generation PPIs	5.94 (30.70)	3.40 (23.06)	1.19 (1.06-1.35)	0.005[a]	
Age ≥75 years old (n = 11,495)					
First-generation PPIs	12.12 (44.79)	7.48 (37.77)	1.16 (1.05-1.27)	0.003[a]	
Second-generation PPIs	8.85 (42.28)	6.18 (34.18)	1.11 (1.00-1.24)	0.049[a]	
Male (n = 14,000)					First-generation PPIs: 0.915 Second-generation PPIs: 0.774
First-generation PPIs	9.86 (39.54)	6.15 (32.03)	1.16 (1.05-1.29)	0.005[a]	
Second-generation PPIs	6.22 (33.65)	4.28 (27.12)	1.12 (0.99-1.27)	0.078	
Female (n = 16,880)					
First-generation PPIs	12.22 (44.78)	6.97 (34.68)	1.16 (1.06-1.27)	0.001[a]	
Second-generation PPIs	7.79 (37.25)	4.63 (28.55)	1.16 (1.04-1.29)	0.007[a]	
Low income (n = 13,085)					First-generation PPIs: 0.692 Second-generation PPIs: 0.217
First-generation PPIs	11.77 (43.14)	6.97 (35.35)	1.17 (1.06-1.29)	0.002[a]	
Second-generation PPIs	7.67 (37.86)	4.29 (27.61)	1.24 (1.11-1.40)	<0.001[a]	
High income (n = 16,880)					
First-generation PPIs	10.62 (41.86)	6.29 (31.94)	1.16 (1.06-1.27)	0.002[a]	
Second-generation PPIs	6.58 (33.78)	4.60 (28.11)	1.07 (0.96-1.20)	0.238	
Urban residents (n = 11,120)					First-generation PPIs: 0.528 Second-generation PPIs: 0.446
First-generation PPIs	9.56 (39.24)	6.15 (32.31)	1.11 (0.99-1.25)	0.071	
Second-generation PPIs	6.49 (33.92)	3.99 (25.39)	1.19 (1.03-1.38)	0.015[a]	
Rural residents (n = 18,845)					
First-generation PPIs	12.04 (44.17)	6.85 (34.14)	1.18 (1.09-1.29)	<0.001[a]	
Second-generation PPIs	7.39 (36.60)	4.75 (29.26)	1.13 (1.02-1.24)	0.018[a]	
Underweight (n = 1,183)					First-generation PPIs: 0.233 Second-generation PPIs: 0.970
First-generation PPIs	15.20 (47.95)	5.53 (31.02)	1.67 (1.19-2.34)	0.003[a]	
Second-generation PPIs	9.51 (46.66)	5.49 (31.66)	1.17 (0.84-1.63)	0.344	
Normal weight (n = 10,805)					
First-generation PPIs	10.73 (40.28)	6.23 (31.90)	1.17 (1.04-1.32)	0.010[a]	
Second-generation PPIs	6.72 (33.63)	4.48 (28.34)	1.09 (0.95-1.26)	0.232	
Overweight (n = 7,819)					
First-generation PPIs	9.66 (36.85)	6.31 (32.77)	1.10 (0.96-1.27)	0.164	

Second-generation PPIs	7.57 (37.16)	4.21 (25.66)	1.27 (1.09-1.49)	0.003[a]	
Obese (n = 10,158)					
First-generation PPIs	12.14 (47.56)	7.31 (35.83)	1.14 (1.02-1.27)	0.018[a]	
Second-generation PPIs	6.73 (34.88)	4.53 (28.59)	1.10 (0.95-1.26)	0.203	
Nonsmokers (n = 22,836)					First-generation PPIs: 0.904 Second-generation PPIs: 0.972
First-generation PPIs	11.14 (42.40)	6.59 (33.23)	1.16 (1.08-1.26)	<0.001[a]	
Second-generation PPIs	6.90 (34.63)	4.38 (27.52)	1.15 (1.05-1.26)	0.003[a]	
Past and current smokers (n = 7,129)					
First-generation PPIs	11.03 (42.52)	6.58 (34.20)	1.14 (0.99-1.30)	0.070	
Second-generation PPIs	7.64 (39.14)	4.74 (29.00)	1.14 (0.97-1.34)	0.123	
Alcohol consumption <1 time a week (n =17,400)					First-generation PPIs: 0.773 Second-generation PPIs: 0.540
First-generation PPIs	11.04 (41.65)	6.52 (33.20)	1.15 (1.06-1.24)	<0.001[a]	
Second-generation PPIs	6.70 (34.95)	3.99 (26.40)	1.15 (1.04-1.26)	0.005[a]	
Alcohol consumption ≥1 time a week (n = 7,849)					
First-generation PPIs	11.42 (45.09)	6.77 (34.20)	1.14 (1.00-1.30)	0.054	
Second-generation PPIs	8.35 (37.90)	5.74 (31.48)	1.07 (0.92-1.25)	0.394	
SBP <140 mmHg and DBP <90 mmHg (n = 20,203)					First-generation PPIs: 0.599 Second-generation PPIs: 0.902
First-generation PPIs	12.00 (43.62)	6.95 (33.86)	1.17 (1.08-1.27)	<0.001[a]	
Second-generation PPIs	7.81 (37.12)	4.85 (28.64)	1.13 (1.03-1.25)	0.010[a]	
SBP ≥140 mmHg and DBP ≥90 mmHg (n = 9,762)					
First-generation PPIs	9.38 (39.91)	5.84 (32.63)	1.10 (0.97-1.24)	0.141	
Second-generation PPIs	5.57 (32.42)	3.65 (26.23)	1.12 (0.96-1.30)	0.143	
Fasting blood glucose <100 mg/dL (n = 17,400)					First-generation PPIs: 0.211 Second-generation PPIs: 0.156
First-generation PPIs	10.29 (39.70)	6.31 (32.75)	1.13 (1.03-1.24)	0.011[a]	
Second-generation PPIs	5.69 (29.70)	3.91 (24.98)	1.10 (0.97-1.24)	0.153	
Fasting blood glucose ≥100 mg/dL (n = 12,565)					
First-generation PPIs	12.10 (45.40)	6.99 (34.49)	1.17 (1.06-1.29)	0.001[a]	
Second-generation PPIs	8.66 (41.45)	5.27 (31.60)	1.14 (1.03-1.27)	0.012[a]	
Total cholesterol level <200 mg/dL (n = 16,447)					First-generation PPIs: 0.869 Second-generation PPIs: 0.879
First-generation PPIs	11.89 (44.67)	7.11 (35.35)	1.15 (1.06-1.25)	0.001*	
Second-generation PPIs	7.34 (37.17)	4.91 (29.80)	1.11 (1.00-1.23)	0.050[a]	
Total cholesterol level ≥200 mg/dL (n = 13,518)					
First-generation PPIs	10.13 (39.33)	5.96 (31.05)	1.14 (1.02-1.27)	0.020[a]	
Second-generation PPIs	6.69 (33.53)	3.93 (25.41)	1.15 (1.01-1.31)	0.034[a]	
Charlson Comorbidity Index = 0 (n = 15,930)					First-generation PPIs: 0.699
First-generation PPIs	8.83 (36.03)	4.80 (26.61)	1.23 (1.09-1.39)	0.001[a]	
Second-generation PPIs	5.95 (31.41)	3.77 (24.70)	1.10 (0.96-1.27)	0.180	

Charlson Comorbidity Index = 1 (n = 5,760)					Second-generation PPIs: 0.102
First-generation PPIs	10.69 (42.27)	8.32 (39.72)	1.06 (0.92-1.21)	0.441	
Second-generation PPIs	6.65 (34.57)	5.04 (29.63)	1.12 (0.94-1.33)	0.218	
Charlson Comorbidity Index ≥2 (n = 8,275)					
First-generation PPIs	13.80 (48.23)	9.35 (41.19)	1.14 (1.03-1.26)	0.009[a]	
Second-generation PPIs	8.47 (40.12)	5.60 (32.86)	1.19 (1.05-1.35)	0.005[a]	
No gastroesophageal reflux disease n = 24,895)					First-generation PPIs: 0.137 Second-generation PPIs: 0.996
First-generation PPIs	4.86 (28.43)	2.74 (21.70)	1.26 (1.13-1.40)	<0.001[a]	
Second-generation PPIs	2.73 (23.02)	1.81 (19.19)	1.15 (1.01-1.31)	0.036[a]	
Gastroesophageal reflux disease (n = 5,070)					
First-generation PPIs	34.94 (69.70)	26.90 (63.67)	1.15 (1.06-1.25)	0.001[a]	
Second-generation PPIs	23.53 (61.16)	18.48 (51.99)	1.19 (1.07-1.31)	0.001[a]	
H2 blocker nonusers (n = 10,069)					First-generation PPIs: 0.037[a] Second-generation PPIs: 0.503
First-generation PPIs	5.02 (34.15)	3.67 (27.03)	1.06 (0.89-1.26)	0.489	
Second-generation PPIs	4.23 (32.42)	2.74 (24.52)	1.16 (0.98-1.37)	0.077	
H2 blocker users (n = 19,896)					
First-generation PPIs	13.24 (44.75)	8.20 (36.44)	1.16 (1.08-1.25)	<0.001[a]	
Second-generation PPIs	8.04 (36.62)	5.42 (29.54)	1.12 (1.02-1.23)	0.014[a]	
No history of head trauma (n = 28,671)					First-generation PPIs: 0.014[a] Second-generation PPIs: 0.001[a]
First-generation PPIs	11.12 (42.39)	6.54 (33.29)	1.19 (1.11-1.27)	<0.001[a]	
Second-generation PPIs	7.21 (36.51)	4.53 (28.22)	1.15 (1.06-1.25)	0.001[a]	
A history of head trauma (n = 1,294)					
First-generation PPIs	11.14 (42.87)	7.94 (38.18)	1.24 (0.94-1.62)	0.124	
Second-generation PPIs	5.24 (22.85)	2.75 (15.96)	1.92 (1.05-3.49)	0.034[a]	
No history of other degenerative diseases of the nervous system (n = 28,933)					First-generation PPIs: <0.001[a] Second-generation PPIs: <0.001[a]
First-generation PPIs	11.11 (42.39)	6.56 (33.37)	1.19 (1.11-1.27)	<0.001[a]	
Second-generation PPIs	6.92 (34.53)	4.51 (28.13)	1.22 (0.88-1.70)	0.230	
A history of other degenerative diseases of the nervous system (n = 1,032)					
First-generation PPIs	11.27 (42.87)	7.86 (37.39)	1.14 (1.04-1.24)	0.003[a]	
Second-generation PPIs	8.66 (46.41)	2.71 (14.62)	1.80 (1.11-2.92)	0.017[a]	

Note: DBP–Diastolic blood pressure; SBP–Systolic blood pressure. [a] Conditional or unconditional logistic regression analysis, Significance at P <0.05; [b] Adjusted for age, sex, income, region of residence, blood pressure, fasting blood glucose, total cholesterol level, obesity, smoking, alcohol consumption, the Charlson comorbidity index, gastroesophageal reflux disease, H2 blocker use, and a history of head trauma or other degenerative diseases of the nervous system.
